# A Paradigm Shift: Arrhythmogenic Cardiomyopathy Is an Inflammatory Disease

**DOI:** 10.3390/cells15100868

**Published:** 2026-05-09

**Authors:** Gallage H. D. N. Ariyaratne, Andrea Villatore, Giovanni Peretto, Stephen P. Chelko

**Affiliations:** 1 Department of Biomedical Sciences, College of Medicine, Florida State University, 1115 West Call Street, Tallahassee, FL 32306, USA; gallage.ariyaratne@med.fsu.edu; 2 Multidisciplinary Disease Unit for Myocarditis and Arrhythmogenic Cardiomyopathies, IRCCS San Raffaele Scientific Institute, 20132 Milan, Italy; 3 Department of Medicine and Surgery, Vita-Salute San Raffaele University, 20132 Milan, Italy

**Keywords:** arrhythmogenic cardiomyopathy, myocarditis, inflammation, desmosome, NFκB

## Abstract

Arrhythmogenic cardiomyopathy (ACM) is a genetic myocardial disorder marked by progressive cardiomyocyte loss, fibro-fatty replacement, ventricular arrhythmias, and risk of sudden cardiac death. Traditionally considered a structural and electrical disease driven by desmosomal dysfunction, emerging evidence redefines ACM as an inflammatory cardiomyopathy in which immune activation plays a central role. This review integrates genetic, molecular, experimental, and clinical data to highlight inflammation as a unifying feature of ACM. Desmosomal gene variants impair cell adhesion and also activate cardiomyocyte-intrinsic inflammatory pathways, including nuclear factor of kappa B (NFκB) and glycogen synthase kinase 3β (GSK3β) signaling, promoting cytokine release, immune cell recruitment, and fibrotic remodeling. Preclinical studies suggest inflammation precedes structural changes, indicating it may be an initiating event rather than a secondary response. Clinical and pathological findings support this model, with inflammatory infiltrates, circulating cytokines, and autoantibodies observed across disease stages. These processes often present as episodic “hot phases” resembling myocarditis, thus complicating diagnosis. The inflammatory landscape involves both innate and adaptive immunity, along with stromal and neuronal remodeling, contributing to arrhythmogenesis through gap junction disruption, calcium-handling abnormalities, and fibrosis. Environmental factors such as exercise, stress, and metabolic disturbances further modulate inflammatory pathways and disease expression. Therapeutically, this evolving perspective supports immunomodulatory approaches, including inhibition of NFκB, GSK3β, and cytokine signaling. Early clinical data on immunosuppressive and cytokine-directed therapies are promising, especially during active inflammatory phases, while gene-based strategies specifically address the underlying genetic defects. In conclusion, ACM should be recognized as an inflammatory cardiomyopathy shaped by interactions between genetic susceptibility and immune dysregulation. Integrating genetic and immunologic profiling may improve diagnosis, risk stratification, and treatment, ultimately leading to refined personalized therapeutic strategies.

## 1. Introduction

Arrhythmogenic cardiomyopathy (ACM) is a genetically and phenotypically heterogeneous myocardial disorder characterized by progressive cardiomyocyte loss, immune cell infiltration, and fibro-fatty replacement of the ventricular myocardium [1,2,3,4,5]. These pathological features create a vulnerable substrate for cardiac dysfunction, malignant ventricular arrhythmias, and sudden cardiac death, with a disproportionate burden in young individuals and athletes [6,7]. Although originally described as a rare inherited heart disease confined to the right ventricle, ACM is now recognized as a spectrum of cardiomyopathic phenotypes, including right-dominant (ARVC), left-dominant (ALVC), and biventricular forms (ACM), reflecting substantial genetic, molecular, and environmental heterogeneity [8,9].

Over the past few decades, advances in next-generation sequencing, experimental in vivo and in vitro disease models, and multimodal cardiac imaging have fundamentally reshaped our understanding of ACM pathogenesis. Desmosomal gene variants (DGVs) remain the most common genetic contributors, accounting for more than 60% of patient cases (Table 1) [10], and growing evidence implicates immune-mediated inflammatory mechanisms in disease onset and progression [11,12]. Other variants in non-desmosomal genes, such as *DES* or *RBM20*, have shown a weaker association with ACM [13,14]. Myocardial inflammation has been recognized for as long as the disease has been known and is a key pathological phenotype of ACM [15]. Generally believed to be manifested by immune cell infiltration [8,9], considering inflammatory infiltrates are observed in 60–88% of patients with ACM and are especially common in patients who died suddenly [16,17], this disease is frequently misdiagnosed as myocarditis [18].

Prior works have shown elevated levels of circulating [22] and myocardial inflammatory cytokines and chemokines from DGV-linked ACM subjects [11,23] that have been observed across disease phases and phenotypes, suggesting that inflammatory processes may represent more than just an epiphenomenon of immune cell-mediated cardiomyocyte injury. In parallel, environmental modifiers such as endurance exercise [24,25,26,27,28,29,30], psychological stress [31,32,33], diet [34,35], alcohol [36], and metabolic dysregulation [37,38,39] have been shown to influence disease penetrance and progression, in part, through modulation of inflammatory processes.

Despite these advances, important gaps remain in defining how genetic susceptibility converges with immune-mediated mechanisms to promote myocardial injury and remodeling, electrical instability, and cardiac dysfunction. In this narrative review, we searched for original clinical and preclinical papers published within PubMed and searched for three specific keywords: “ACM”, “inflammation” and “immunity.” We aimed to integrate genetic, molecular, experimental, and clinical evidence to highlight the undeniable role of myocardial inflammation as a recurrent, unifying feature of ACM pathobiology. Additionally, we discuss the consequences that myocardial inflammation imparts on disease classification, risk stratification, and the development of novel therapeutic strategies to mitigate immune-mediated myocardial inflammation.

## 2. Race to Discovery: Myocardial Inflammation as a Key Pathological Phenotype in ACM

**Figure 1:**
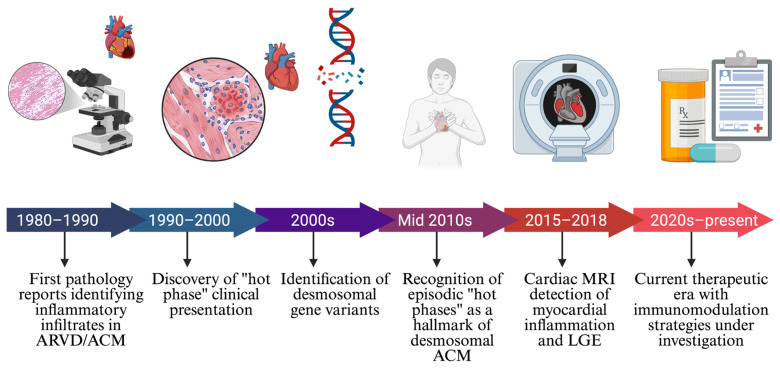
Historical timeline of myocardial inflammation in ACM. Chronological overview of key milestones in the recognition and characterization of inflammation in ACM. Initial pathological observations of inflammatory infiltrates in ARVD/ACM (1980–1990) were followed by the clinical description of the “hot phase” phenotype (1990–2000) and the identification of causative DGVs (2000s). The mid-2010s brought recognition of episodic hot phases as a hallmark of desmosomal ACM, while advances in cardiac MRI and LGE-MRI enabled non-invasive detection of myocardial inflammation (2015–2018). The current era (2020s–present) is defined by active investigation of immunomodulatory therapeutic strategies.

Myocardial inflammation is a pathological hallmark of ACM since its first discovery in 1982 (Figure 1) [15,40]. Histological findings are reminiscent of acute myocarditis and are often reflective of an active “hot phase” of ACM that is associated with accelerated disease progression [40,41]. Inflammatory infiltrates, consistent with T lymphocytes and monocytes, are frequently reported in pathological samples from both ventricles of patients with ACM [15,17,42,43], often fulfilling both the Dallas and European Society of Cardiology (ESC) criteria for acute myocarditis [44,45], with or without the detection of cardiac viral genomes. However, recent works have demonstrated CCR2^+^ macrophages are early and persistent drivers of cardiac inflammation in ACM [12].

In parallel, elevated sera levels of anti-heart (e.g., anti-α- and β-myosin-heavy chains), anti-intercalated disc, and anti-desmoglein-2 autoantibodies have been reported in ACM and are strongly correlated with gap junction dysfunction and ventricular arrhythmias (VAs) [46,47,48,49]. Furthermore, elevated levels of circulating cytokines [19] and cardiac troponin T (cTnT) [50,51,52] are indicative of active myocardial inflammation and injury. These inflammatory and injurious phases are often detected in ACM patients via minimally invasive advanced imaging techniques, such as cardiac magnetic resonance (CMR) [45], late gadolinium enhancement (LGE) MRI, and ^18^F-fluorodeoxyglucose (FDG) positron emission tomography (PET) [53,54]. Despite its frequent misdiagnosis as acute myocarditis, this interpretation has serially been described as a clinical manifestation of ACM or, at least in part, as a progressive complication during its natural history [41,45,50,55]. The so-called “hot phase” of desmosomal ACM occurs more frequently in young individuals and women and is characterized by chest pain, peaks in serum cTnT, and mildly reduced or preserved cardiac function [56,57,58]. However, despite a presumed benign presentation, DGV-positive patients with myocarditis-like phenotypes are at increased risk of major VAs and heart failure, as compared to DGV-negative patients [56,57,58]. These clinical similarities have led to the frequent misdiagnosis of ACM as myocarditis and the potential underestimation of ACM disease prevalence, thus putting patients at risk as well.

A recent study [52] identified the following “red flags” enabling the recognition of the “hot phase” of ACM over classic acute myocarditis: (i) a familial history of cardiomyopathy, myocarditis, and/or sudden cardiac death/arrest (SCD/A); (ii) recurrent spikes of serum cTnT; (iii) persistence of left ventricular dysfunction during follow-up; (iv) evidence of RV disease via imaging; (v) LGE enhancement with ring-like patterns or (vi) LGE showing either persistence or extension during follow-up; (vii) low QRS voltage on ECG; (viii) life-threatening VAs at age <45 years; (ix) persistence of frequent (>1000/24 h) ventricular ectopy; and (x) recurrent non-sustained VAs. Whenever a DGV is identified by genetic testing, more focused follow-up and risk stratification strategies should be pursued as compared with classic acute myocarditis, as well as cascade genetic and clinical screening in first-degree family members.

Additional studies are needed to further refine the differential diagnosis and optimize the clinical management of patients with active hot phases.

## 3. Pathology: Histological Markers of Myocardial Inflammation

Recent developments in our understanding of the causes of myocardial inflammation in ACM have been reframed from a consequence of myocyte cell death, now to intrinsic factors originating from cardiomyocytes that harbor DGVs. These pivotal works, utilizing in vivo and in vitro models of ACM, human biopsies, state-of-the-art techniques (e.g., snRNAseq, CITseq, and spatial transcriptomics), and therapeutic interventions, have been essential in elucidating disease-causing culprits. This has moved the field to the characterization of ACM as an inflammatory cardiomyopathy, stemming from desmosomal disruption. In essence, this re-evaluation underscores the role of inflammation as a fundamental component of ACM, rather than a consequence of it, that affects all ventricular-specific subtypes.

### 3.1. Inflammation as the Initiating Event in Preclinical Animal Models

Multiple desmoglein-2 (*Dsg2*) mutant mouse models have been instrumental in elucidating the inflammatory mechanisms underlying ACM pathogenesis, though important distinctions exist regarding mutation type, tissue specificity, and clinical relevance.

#### 3.1.1. Desmoglein-2 Mutant Models of ACM

- Cardiac-Restricted *Dsg2*^−/−^ Model

By employing αMHC-Cre-mediated recombination, Ng KE and colleagues [59] successfully generated a mouse model with *Dsg2* deleted solely in cardiomyocytes, resulting in a cardiac-restricted homozygous *Dsg2*-null (αMHC-*Dsg2*^−/−^) mouse line. Transcriptomic analysis was performed at postnatal day 14 (P14) before evidence of fibrotic remodeling to assess the primary mechanisms of inflammation activated as a consequence of desmosomal disruption. Bulk RNAseq identified inflammatory signatures, including TNFα and IL-6, and activation of the iRhom2/ADAM17 pathway, which were all significantly elevated in αMHC-*Dsg2*^−/−^ hearts [59]. At this early timepoint, cardiomyocyte apoptosis was significantly increased and thus proposed as the trigger leading to inflammatory immune cell recruitment and subsequent cardiac remodeling [59]. Myocardial lesions displayed increased numbers of infiltrating CD45^+^ leukocytes, primarily F4/80^+^ macrophages [59]. While no collagen deposition was detected in P14 hearts, numerous pro-fibrotic mediators were significantly upregulated, including *Col1a1*, *Col3a1*, and *Ctgf* [59]. However, the absence of a germline mutation in this mouse model limits its ability to accurately reflect the genetic origins of human ACM.

b. Germline *Dsg2*^mut/mut^ Model

The homozygous desmoglein-2 mutant (*Dsg2*^mut/mut^) mouse model of ACM has been a critical preclinical model in the investigation of the inflammatory processes that contribute to disease pathogenesis in ACM [11,12,29,31,60,61,62]. The germline *Dsg2*^mut/mut^ mouse model developed by Chelko SP and colleagues represents a more genetically relevant system for studying ACM pathogenesis, as the *Dsg2* mutation is germline, mirroring the pattern of inheritance observed in patients [12,60]. This model exhibits progressive myocardial inflammation, fibrotic remodeling, and ventricular dysfunction and arrhythmias. These mice develop disease phenotypes in a phase-specific manner, as observed in patients with ACM (i.e., “concealed phase” followed by episodic “hot phases”). Furthermore, these authors demonstrated myocardial inflammation arises as an intrinsic consequence of the *Dsg2* mutation [11,12], reflecting mutation-driven inflammatory remodeling.

Mechanistically, the inflammatory phenotype is characterized by accumulation of CCR2^+^ monocyte-derived macrophages within the myocardium [12]. Cardiomyocyte-intrinsic activation of nuclear factor kappa-B (NFκB) signaling plays a central role in promoting myocardial injury and arrhythmogenesis through recruitment of these inflammatory macrophages [12]. Multi-omic analyses, including single-nucleus RNA sequencing (snRNAseq) and cellular indexing of transcriptomes and epitopes sequencing (CITE-Seq), demonstrated inflammatory transcriptional reprogramming across cardiomyocytes, fibroblasts, and both myeloid and lymphoid lineage cells, indicating extensive immune–stromal crosstalk during disease progression [12]. These outcomes further stress the importance of germline mutations rather than cardiomyocyte-specific DGVs for in vivo disease modeling.

Further analyses identified expansion of inflammatory macrophage subsets interacting with activated fibroblast populations, forming inflammatory–fibrotic niches that promote cardiac remodeling [12]. Direct pharmacologic inhibition of the master regulator of inflammatory signaling, NFκB, via the small molecule Bay 11-7082, normalized myocardial levels of several pro-inflammatory cytokines (e.g., IL-1β, IFN-γ, and IL-12), chemokines (e.g., LIX and CCL2), and fibrokines (e.g., OPN and POSTN) with ameliorated myocardial injury and improvement in cardiac function [12].

c. Cardiomyocyte-Specific *Dsg2*^mut/mut^ Model

A separate cardiomyocyte-specific *Dsg2*^mut/mut^ model has also been reported [63]. Although this model uses the same *Dsg2*^mut/mut^ nomenclature as reported above, it represents a distinct system in which the mutation is restricted to cardiomyocytes rather than germline presence (*Myh6*-cre mouse mated with *Dsg2*-loxP mouse) [63]. Consequently, while useful for dissecting cardiomyocyte-specific mechanisms, this model does not fully reproduce the germline mutational context of human ACM, limiting its translational relevance compared with germline models. That said, this model recapitulates key ACM phenotypes, including progressive ventricular dysfunction, cardiomyocyte loss, inflammatory cell infiltration, and fibrotic remodeling, supporting a primary role for cardiomyocyte injury in driving disease progression [63]. Furthermore, pharmacological inhibition of NOD-like receptor protein 3 (NLRP3) mitigated ventricular dilation and dysfunction [63].

d. Comparative Implications

Collectively, these *Dsg2*-mutant mouse models provide complementary insights into ACM pathogenesis. Cardiac-restricted models have been instrumental for identifying early inflammatory signaling pathways triggered by cardiomyocyte injury and/or cell death. However, the germline *Dsg2*^mut/mut^ model developed by Chelko SP and colleagues more accurately reflects the inherited genetic context of ACM [12,60], making it particularly valuable for studying mutation-driven inflammatory remodeling and arrhythmogenesis. Importantly, findings from this model demonstrate that myocardial inflammation arises intrinsically (i.e., from cardiomyocytes) due to the presence of a desmosomal mutation, rather than solely from infiltrative immune cells, supporting the concept that inflammation is a primary driver of disease progression and an emerging therapeutic target in ACM.

#### 3.1.2. Plakophilin-2 Experimental Model of ACM

*PKP2* is the most frequently mutated desmosomal gene found in patients with ACM who are DGV-positive, despite the higher risk of developing heart failure in patients harboring *DSG2* variants, as demonstrated in the multicenter study by Hermida A and colleagues [64].

The cardiomyocyte-specific, tamoxifen-inducible *Pkp2* knock-out (*Pkp2*-cKO) mouse model was originally described by Cerrone M and colleagues [65], who demonstrated that loss of *PKP2* exclusively in adult cardiomyocytes is sufficient to cause ACM of right ventricular predominance, transcriptional downregulation of key calcium-handling genes (e.g., *Ryr2*, *Ank2*, *Cacna1c*, and *Trdn*), disruption of intracellular calcium homeostasis, and catecholamine-induced ventricular arrhythmias [65]. Of note, the latter phenotype was suppressed by flecainide treatment. Additionally, *Pkp2*-cKO induces pro-inflammatory senescence in non-myocyte cells and premature cardiac aging [66].

Studies conducted by Wu I and colleagues [67] demonstrated that c*Pkp2*-cKO mice develop progressive ventricular dysfunction and structural remodeling. In a separate study utilizing the same *Pkp2*-cKO mouse model as above, molecular studies by Pérez-Hernández M and colleagues [68] demonstrated cardiomyocyte-intrinsic stress responses, including increased reactive oxygen species production, loss of nuclear envelope integrity, DNA damage, and myocyte cell death. Notably, these responses were exacerbated by exercise or β-adrenergic stimulation, supporting the framework that cardiomyocyte injury initiates inflammatory disease progression and the role of environmental factors as inducers of disease penetrance and severity [68].

In the study by Wu I and colleagues, restoration of *PKP2* expression via AAV9-mediated gene therapy improved cardiac function and survival in *Pkp2*-cKO mice [67]. Finally, the lasting effect of this therapeutic correction on transcriptional networks, specifically those impacting genes involved in sarcomere structure and calcium regulation, further supports that desmosomal dysfunction is the primary instigator of these aberrant fibro-inflammatory, structural, and calcium-handling cascades [67].

#### 3.1.3. Desmoplakin Experimental Models of ACM

Desmoplakin (*DSP*) variants are associated with left-dominant ACM, and common presentations are characterized by chest pain, arrhythmias, and episodic hot phases associated with LGE-MRI of the left ventricular free wall [69]. The latter characteristic often leads to its misdiagnosis as acute myocarditis [18,52]. To model the cardiomyocyte-intrinsic aspects of *DSP*-associated innate immune activation, Selgrade DF and colleagues generated two distinct engineered heart tissue (EHT) systems: (a) patient-derived EHTs harboring heterozygous *DSP* truncating variants (*DSP*-tvs; [i] *DSP* p.E1597X and [ii] *DSP* p.R1951X), and (b) a more severe gene-edited homozygous deletion line (*DSP*^−/−^) generated from a healthy control iPSC-CM line using CRISPR/Cas9 [70].

At baseline, *DSP*^−/−^ EHTs exhibit a transcriptomic signature of innate immune activation, mirrored by elevated concentrations of multiple cytokines, including IL-1β, IL-6, IL-8, IFN-γ, IL-17, and IL-23, which were detected in the EHT cell culture media; thus, they are of cardiomyocyte origin rather than from immune cells [70]. In contrast, heterozygous *DSP*-tv EHTs exhibited only minimal baseline contractile deficits, though they exhibited elevated innate immune activation relative to healthy controls, reflected by increased IL-1β secretion into cell culture media upon Toll-like receptor (TLR) stimulation via exogenous application of HMGB1. Importantly, both *DSP*^−/−^ and *DSP*-tv EHTs demonstrated heightened sensitivity to pan-TLR stimulation compared to respective controls, though the contractile dysfunction was more severe in *DSP*^−/−^ EHTs [70]. A finding unique to heterozygous *DSP*-tv EHTs was the reduction in contractile reserve that was unmasked specifically under conditions of mechanical strain, consistent with the clinically observed concealed phase that precedes overt structural disease in heterozygous *DSP* variant carriers.

Anti-inflammatory strategies, including colchicine, a broad anti-inflammatory agent, or NFκB inhibitors, effectively rescue these force deficits. Additionally, adenine base editing to genomically correct the *DSP* p.R1951X pathogenic variant restored *DSP* expression and reduced the secretion of multiple pro-inflammatory cytokines into culture media [70]. Of monumental importance, genetic correction of *DSP* p.R1951X was accompanied by a substantial reduction in *NFκB* mRNA expression, further suggesting that the mere presence of a desmosomal gene variant drives myocardial inflammation rather than being a consequence of infiltrating immune cells.

The *Dsp^S311A^* knock-in mouse model developed by Guazzo A and colleagues [71], which carries a mutation analogous to the human *DSP* p.S299R variant, recapitulates *DSP*-linked cardiomyopathy phenotypes in a gene dose-dependent manner. More specifically, homozygous *Dsp^S311A/S311A^* mice developed early biventricular dysfunction, fibrosis, inflammation, arrhythmias, and cutaneous defects, similar to Carvajal syndrome, while heterozygous *Dsp^WT/S311A^* mice exhibited dominant *DSP*-related cardiomyopathy features, including fibrosis, apoptosis, inflammation, and electrical instability [71]. Desmosomal remodeling is evident in both *Dsp^WT/S311A^* and *Dsp^S311A/S311A^* mice, such as connexin-43 (Cx43) mislocalization as early as 1 month of age, whereas reduced β-catenin nuclear translocation and DSP/DSG2 protein levels were exclusively found in *Dsp^S311A/S311A^* mice. Spontaneous arrhythmias and electrical instability manifested early in both *Dsp^WT/S311A^* and *Dsp^S311A/S311A^* mice in response to forced exercise (via treadmill) significantly accelerated disease features and premature death. This model, as described by Guazzo A and colleagues, uniquely combines arrhythmias, inflammation, and extra-cardiac features, providing an important in vivo system to study *DSP*-related mechanisms and evaluate therapeutic interventions aimed at preventing SCD [71].

#### 3.1.4. Temporal Dynamics in Mouse Models

Desmosomal-linked models of ACM have collectively proposed a temporal sequence wherein inflammatory signaling precedes and drives structural remodeling. Critically, snRNA-seq analyses revealed that cardiomyocyte-intrinsic NFκB activation not only recruits CCR2^+^ inflammatory macrophages but also coincides with depletion of cardioprotective LYVE-1^+^ resident macrophages, a shift in the immune landscape that drives negative inotropic effects in viable myocardium even before extensive replacement fibrosis [12]. This temporal progression of cardiomyocyte stress response, followed by inflammation and culminating in extensive ventricular fibrosis, highlights the central and episodic role of inflammation in ACM disease onset and progression [12].

### 3.2. Human Pathological Reports of Myocardial Inflammation in ACM

Endomyocardial biopsies, post-mortem reports, and/or explanted hearts from patients with ACM have revealed that pro-inflammatory infiltrates invariably accompany myocyte depletion and fibro-fatty remodeling of the myocardium, specifically in areas of active cardiac injury [72]. Uniquely, GSK3β localization at the intercalated disc (ID) was observed in ACM hearts. This finding was in stark contrast to other inflammatory heart diseases, such as giant cell myocarditis and cardiac sarcoidosis, where GSK3β localization was predominantly cytoplasmic [60]. While not a diagnostic tool for ACM, GSK3β localization at the myocyte–myocyte ID may provide further pathological evidence of the unique contributions GSK3β imparts in ACM vs. myocarditis and/or other forms of heart disease where myocardial inflammation is a key pathological phenotype.

Given that endomyocardial biopsies often reveal inflammatory infiltrates in patients with ACM, a differential diagnosis can be challenging between ACM and myocarditis [18,52]. However, uniquely distinguishing features have been noted in ACM hearts, such as loss of junctional plakoglobin (JUP), Cx43 [73], and SAP97 [74] at the ID, in conjunction with GSK3β redistribution to the ID [60], demonstrating that desmosomal disruption imparts uniquely specific pathological characteristics in ACM. Clinical findings often report the so-called “hot phases” of ACM, where episodic bouts of myocardial inflammation, chest pain, and elevated troponin levels occur in genetically predisposed individuals, particularly those harboring *DSP* variants [70].

#### Genotype–Phenotype Correlations

Left-dominant ACM: genotype has a significant impact on the structural and inflammatory trajectory of ACM. *DSP*-linked ACM is strongly associated with a myocardial fibro-inflammatory phenotype and a higher frequency of episodic “hot phases,” typically manifesting in the left ventricle and frequently observed via LGE-MRI [52,70]. Additionally, *DSG2* variants are also characterized by extensive left ventricular involvement, demonstrating substantial fibro-inflammatory remodeling [62], as well as an unrecognized multicellular pathology involving the autonomic nervous system [62]. Recent work by Vanaja IP et al. demonstrated *DSG2* is expressed in human cardiac sympathetic neurons, and its deficiency leads to massive subepicardial sympathetic hyperinnervation that precedes overt structural remodeling [62], potentially serving as an early arrhythmic substrate.

Right-dominant ACM: Patients with *PKP2* variants classically present with right ventricular dominant disease and exhibit a unique vulnerability to environmental perpetrators [32]. Clinical data indicates that physical exercise, specifically endurance and high-intensity, significantly correlates with disease progression and arrhythmias in *PKP2* mutation carriers [24], as well as in *DSP* carriers and gene-elusive ACM subjects [25,27], suggesting a gene–environment interaction. In *Pkp2*-cKO mice, exercise enhanced cardiac dysfunction, but its effect was attenuated by exercise pre-training [30].

Collectively, these prior findings demonstrate local myocardial inflammation, and pro-inflammatory infiltrates are not only pathological hallmarks of ACM but are also primary drivers of disease.

### 3.3. The Cellular and Molecular Landscape of Inflammation in ACM

The inflammatory landscape of ACM represents a paradigm shift from a purely myocyte-centric view to a multicellular and multiorgan inflammatory disease. Rather than serving simply as a marker of end-stage tissue damage, immune activation occurs prior to overt structural remodeling and mechanistically drives disease progression.

#### 3.3.1. Pro-Inflammatory Cytokines and Chemokines

Multiple pro-inflammatory cytokines and chemokines have been shown to be upregulated early in ACM pathogenesis. In *Dsg2*^mut/mut^ mice, myocardial levels of IL-1β, IFN-γ, IL-12, LIX (CXCL5), and Osteopontin (OPN) are substantially elevated. Notably, LIX and OPN levels show a significant inverse correlation with cardiac function and a positive correlation with myocardial fibrosis [60], suggesting their utility as biomarkers for inflammatory disease severity. Furthermore, environmental modifiers can amplify cytokine and fibrokine burden, such as the recent work by Centner A et al. that demonstrated high-fat diet (HFD) exposure in *Dsg2*^mut/mut^ mice dramatically elevates myocardial levels of adiponectin (AdipoQ) and fibroblast growth factor 1 (FGF1) [35].

Chemokine signaling, particularly the CCR2/CCL2 (MCP-1) axis, acts as a primary driver for recruiting monocyte-derived macrophages to the myocardium. Transcriptomic data confirms this upregulation precedes structural damage, facilitating the early formation of pathological “inflammatory–fibrotic niches” [12,59].

#### 3.3.2. The Multicellular Infiltrate

Recent work has identified a complex interplay between innate, adaptive, and extra-cardiac cell populations within these inflammatory–fibrotic niches. Within the innate immune compartment, cardiomyocyte-intrinsic signaling orchestrates the recruitment of CCR2^+^ monocyte-derived macrophages. These infiltrating macrophages subsequently contributed to the expansion of fibroblasts and the elevation of heart failure-related markers (*Ankrd1* and *Xirp2*) in neighboring myocytes [12].

The adaptive immune system also plays a critical role in the cellular microenvironment that composes the hearts of ACM subjects. Systems-level transcriptomic analyses of end-stage human ACM hearts identified a complex landscape enriched with specific T-cell subsets, including Th1, Th2, and Treg populations. Notably, a significant positive linear correlation existed between the degree of total immune cell infiltration and extracellular matrix organization enrichment score, highlighting the interdependence of immune activation and fibrotic remodeling in disease progression [75].

#### 3.3.3. Pathological Signaling Mediators

**Figure 2:**
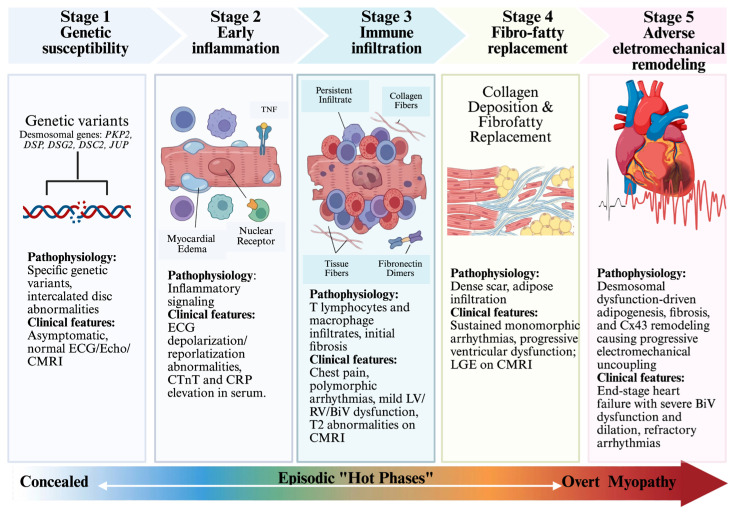
Stages of ACM disease progression. Five-stage model of ACM progression from concealed to clinically evident disease. Stage 1 (genetic susceptibility): DGVs disrupt intercalated disc integrity in otherwise asymptomatic individuals. Stage 2 (early inflammation): inflammatory signaling manifests as ECG abnormalities and elevated serum biomarkers. Stage 3 (immune infiltration): T lymphocyte and macrophage infiltration with initial fibrosis, presenting with chest pain and mild ventricular dysfunction. Stage 4 (fibro-fatty replacement): dense fibrotic scars and adipose infiltration drive sustained VAs and progressive ventricular dysfunction detectable by LGE on cMRI. Stage 5 (adverse electromechanical remodeling): desmosomal dysfunction-driven adipogenesis, fibrosis, and progressive electromechanical uncoupling, culminating in end-stage heart failure with biventricular dysfunction and refractory arrhythmias.

The orchestration of this multicellular inflammatory response is heavily dependent on master regulatory pathways, notably the canonical NFκB and Wnt/β-catenin pathways. Canonical NFκB activation serves as an early programmatic driver of myocardial injury and heightened reactivity to innate immune triggers. Concurrently, the pathognomonic redistribution of GSK3β appears to be uniquely specific to ACM. Targeting specific mediators within these pathways, whether through GSK3β inhibition (e.g., Tideglusib) [76] or NFκB inhibition, has been shown to restore cardiac function and attenuate disease-associated remodeling in experimental models of ACM, further supporting mediators of inflammation as therapeutic targets. However, it should be noted that long-term inhibition of GSK3β and NFκB would undoubtedly lead to an elevated risk of developing cancer and suppression of the innate immune response, respectively (Figure 2).

## 4. Cell Autonomous Mechanisms in Arrhythmogenic Cardiomyopathy

**Figure 3:**
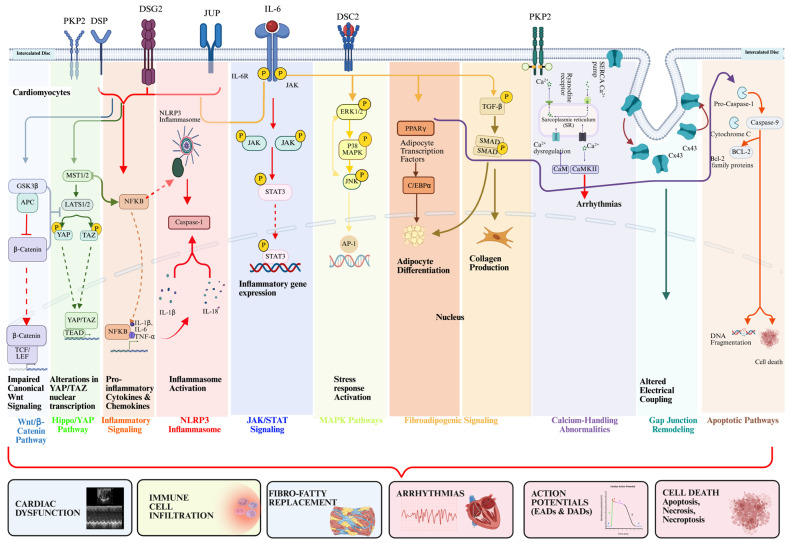
Signaling pathways underlying ACM pathogenesis. Schematic representation of key molecular signaling cascades activated by DGVs in cardiomyocytes. Collectively, these pathways converge to produce the hallmark features of ACM: cardiac dysfunction, immune cell infiltration, fibro-fatty replacement, arrhythmias, abnormal action potentials, and cardiomyocyte death.

ACM is increasingly recognized as an inflammatory disease where cardiomyocytes harboring DGVs lead to NFκB activation, propagating inflammatory cascades that lead to ACM disease onset and progression [11]. Perturbations of myocyte–myocyte ID junctions are a consequence of desmosomal disruption and affect regulatory signaling hubs, such as NFκB signaling, the Wnt/β-catenin and GSK3β pathways, Hippo/YAP signaling, and a variety of cell death pathways, culminating in aberrant signaling programs that lead to mechanical and electrical instability, mitochondrial dysfunction, endoplasmic reticulum (ER) stress, and aberrant mechanotransduction within cardiomyocytes [11,12,29,60,77]. These aberrant signaling pathways (Figure 3) lead to chemokine and cytokine production, facilitating local inflammation and recruitment of inflammatory cells to the heart, further amplifying arrhythmogenesis and clinical manifestations [11,12]. Pharmacological and genetic approaches targeting cell-autonomous programs within cardiomyocytes have highlighted therapeutic avenues to block early abnormal signaling cascades and are detailed below with additional information on preclinical data and/or clinical trials when available [11,12,60].

### 4.1. NFκB Signaling in ACM

NFκB is a transcription factor that regulates the expression of numerous inflammatory molecules and has been shown to be activated early in ACM pathogenesis within cardiomyocytes, representing a key therapeutic target. Increased NFκB activation (evidenced by RelA/p65 nuclear localization) is observed in iPSC-CMs generated from patients with ACM, as well as in in vitro and in vivo models of ACM. This intrinsic activation is demonstrated by increased NFκB nuclear localization and elevated expression of NFκB-mediated gene transcripts, independent of exogenous inflammatory stimuli [11,12].

Downstream NFκB activation leads to the expression of inflammatory chemokines (e.g., LIX, CCL2, CXCL10) and cytokines (e.g., IL-1β, IL-6, TNFα), which have been tied to the recruitment of inflammatory cells and maladaptive tissue remodeling [11]. Further analysis using single-nucleus RNA sequencing (snRNA-seq) has shown that pathways enriched in NFκB family genes are upregulated in *Dsg2*-mutant cardiomyocytes, demonstrating an essential role for NFκB in ACM-associated innate immune signaling [12]. NFκB is activated downstream of desmosome disruption through increased mechanical stretch, endoplasmic reticulum (ER) stress, reactive oxygen species (ROS), and the autocrine production of cytokines [78].

NFκB has multiple downstream functions that lead to the recruitment of CCR2^+^ monocyte-derived macrophages to the hearts of ACM subjects and the subsequent remodeling of ion channels and gap junctions. These collective changes lead to slowed conduction, arrhythmias, and cardiac dysfunction [11,12]. The small-molecule inhibitor of NFκB, Bay 11-7082, has shown to mitigate inflammation, fibrosis, cardiac dysfunction, and arrhythmogenesis in preclinical models [11].

### 4.2. Wnt/β-Catenin and Hippo/YAP Signaling

#### 4.2.1. Canonical Wnt/β-Catenin Signaling Pathway

Canonical Wnt signaling stabilizes β-catenin by inhibiting its phosphorylation through the GSK3β-APC-Axin destruction complex, allowing for β-catenin to translocate to the nucleus and mediate TCF/LEF-induced transcription to promote tissue homeostasis. In a landmark study by Garcia-Gras E et al. [77], these authors demonstrated that suppression of canonical Wnt/β-catenin signaling leads to ACM, prominently through competition with nuclear localization of JUP, rather than through β-catenin degradation [77,79,80].

Decreased Wnt/β-catenin signaling promotes fibro-adipogenic pathways that can direct progenitor cells into becoming adipocytes. In experimental models, extracellular vesicles engineered to deliver or augment β-catenin levels have been shown to attenuate inflammation and fibrosis in *Dsg2*-mutant mice [81]. Preclinical studies suggest Wnt pathway augmentation may be a disease-modifying target in ACM; however, clinical translation remains unproven.

#### 4.2.2. Hippo/YAP Signaling Pathway

Activation of Hippo pathway kinases (MST1/2 and LATS1/2) leads to the phosphorylation and cytoplasmic retention of YAP/TAZ. When Hippo signaling is suppressed, YAP and TAZ translocate to the nucleus and interact with TEAD co-transcription factors to activate genes involved in cell survival and proliferation [79,82].

Conversely, in ACM, prior studies have demonstrated aberrant Hippo pathway activation and subsequent YAP inactivation [79]. The loss of nuclear YAP in conjunction with Wnt/β-catenin suppression further drives adipogenesis and maladaptive fibrotic repair [79,80]. Therefore, therapeutic strategies would logically aim to restore YAP activity or dampen upstream Hippo kinase activity, rather than inhibiting YAP effectors. While manipulating these interconnected pathways represents a compelling hypothesis for future research, the clinical efficacy of such targeted therapies for arrhythmia prevention in patients with ACM remains speculative.

### 4.3. GSK3β and Fibro-adipogenic Signaling

GSK3β serves as a critical point of convergence among Wnt, NFκB, and adipogenic signaling pathways, representing a promising therapeutic target based on robust preclinical data. As previously described, GSK3β phosphorylates β-catenin, targeting it for proteasomal degradation, suppressing canonical Wnt signaling. Aberrant GSK3β activity in ACM is also linked to the deleterious activation of NFκB [11,12,60]. Together, the suppression of Wnt and overactivation of NFκB drive pro-inflammatory, adipogenic, and fibrotic gene expression programs [60].

Pharmacological inhibition of GSK3β (e.g., SB216763) prevented myocyte injury and cardiac dysfunction in two murine models of ACM (*Dsg2*^mut/mut^ and *JUP*^2157del2^) at rest and in response to exercise, where SB216763-treated mice demonstrated improvements in ventricular ectopy and function, myocardial fibrosis and inflammation, and normalization of ID proteins, and, most importantly, increased survival [60]. Building on these findings, Tideglusib, a repurposed oral GSK3β inhibitor, is currently being evaluated in the Phase 2 TaRGET clinical trial to determine its efficacy in reducing ectopic burden in genotype-positive patients with ACM, with secondary endpoints evaluating changes in ventricular strain, bouts of sustained ventricular tachycardia, and appropriate and inappropriate discharges.

### 4.4. Adipogenic and TGF-β/SMAD Signaling

Building upon Wnt suppression mechanisms (Section 4.2.1 and Section 4.2.2), loss of canonical Wnt signaling can lead to PPARγ and C/EBPα-mediated adipogenesis (i.e., transcription and cellular differentiation). Recent evidence implicates fibro-adipogenic progenitors and epicardial-derived cells as primary sources, and they typically show an epicardial to endocardial migration [77,79,83,84].

TGF-β1 activates SMAD2/3 signaling, driving pro-fibrotic gene expression and extracellular matrix deposition in cardiac fibroblasts [85]. Crosstalk between inflammatory pathways and TGF-β may explain the heterogeneous patchiness of fibro-fatty replacement [86]. PPARγ and TGFβ inhibitors have also shown promise in animal models of ACM, while direct PPARγ agonism (via Rosiglitazone) has demonstrated increased mortality in *Dsg2*-mutant mice [87,88].

### 4.5. Calcium-Handling Abnormalities

Abnormal calcium handling has been linked to molecular and cellular alterations in ACM with elevated arrhythmogenicity. Dysfunction of RyR2 calcium channels and SERCA2a pumps drives increased diastolic calcium leak and spontaneous calcium transients, triggers for arrhythmias. Furthermore, inflammatory cascades (Section 4.1) have been shown to contribute to calcium mishandling through multiple mechanisms, such as (i) cytokine-induced ion channel dysfunction, (ii) elevated ROS levels that originate from infiltrating macrophages, (iii) chronic CaMKII activation, (iv) phosphorylation of RyR2, and (v) increased calcium leak. Oxidative stress further promotes direct RyR2 modification via oxidation of cysteine residues [89], producing S-nitrosylation, S-glutathionylation, and intersubunit disulfide cross-linking, collectively increasing channel open probability and augmenting diastolic sarcoplasmic reticulum (SR) calcium leak that drives arrhythmogenesis. Notably, ROS-mediated oxidation of CaMKII at methionine residues 281/282 sustains its activation independent of calcium–calmodulin, leading to persistent RyR2 phosphorylation at serine 2814, an effect that acts additively with direct cysteine oxidation to further destabilize SR calcium homeostasis [89].

Importantly, perturbed calcium handling often precedes structural remodeling, suggesting this is a primary pathogenic event predisposing the hearts to malignant VAs [30]. Flecainide, a sodium channel blocker, has also shown promising anti-inflammatory and antiarrhythmic properties via RyR2 modulation [90,91].

### 4.6. Gap Junction Remodeling and Electrical Coupling

Disruption to the myocyte–myocyte ID is a key pathological hallmark of ACM, leading to impaired electrical communication between cardiomyocytes. In addition, Cx43 is a critical gap junction protein, and its decreased expression, aberrant phosphorylation [92], and absence at the ID are frequent disease characteristics in ACM. This impairs electrical coupling, substantially decreasing conduction velocity and promoting reentrant circuits [93]. Notably, electrophysiological defects precede structural changes, suggesting Cx43 pathology is an additional initiator of ACM disease onset and development [93].

Mechanistically, desmosomal protein deficiencies lead to Cx43 redistribution [94], gap junction remodeling, and increased arrhythmogenesis [95]. A recent study demonstrated that gene therapy delivered using an exogenous AAV9-GJA1-20k, a 20 kDa isoform of Cx43, successfully rescued gap junction distribution and reduced VAs in an ACM mouse model [96], further supporting the critical role, or lack thereof, of Cx43 in ACM.

### 4.7. Apoptotic and Necrotic Signaling Pathways

Cardiomyocyte death through apoptotic and necrotic pathways drives progressive myocardial loss and fibro-fatty replacement in ACM. Histopathological analyses using a variety of immunohistochemical techniques have demonstrated increased apoptotic cardiomyocyte death in ACM, with apoptotic indices reaching approximately 24% in early symptomatic patients [97,98], suggesting myocardial destruction may be episodic rather than a gradual loss.

A critical exercise-induced, mitochondrial-mediated pathway was recently uncovered, involving the calcium-dependent cysteine protease, calpain-1 (CAPN1), where CAPN1 cleaves the mitochondrial-bound apoptosis-inducing factor (AIF) [29]. Truncated AIF translocates to the nucleus, inducing caspase-independent DNA fragmentation and cell death, a specialized necroptotic cell death program. This is potentiated by mitochondrial oxidative stress due to depleted thioredoxin-2 (TRX2) and TRX2 reductase (TXNRD2) stores, leading to impaired ROS buffering [29]. It should be noted that *Trx2* and *Txnrd2* are Wnt/β-catenin-induced gene transcripts, thus additionally highlighting suppression of the Wnt/β-catenin pathway in ACM pathogenesis. Additional mitochondrial apoptotic markers include CPP-32 and BAX with absent BCL-2 expression [98]. Necrosis initiates myocardial injury, triggering inflammation, calcification, and fibrotic replacement [99].

### 4.8. Non-Myocyte Mechanisms and Intercellular Crosstalk

ACM pathophysiology extends beyond cardiomyocytes to involve cardiac stromal cells, immune cells, and endothelial cells. Cardiac mesenchymal stromal cells (c-MSCs) that harbor DGVs can promote fibro-adipogenic differentiation through bidirectional paracrine signaling [100]. The DLK1-NOTCH pathway is critical for this differentiation, as DLK1 suppresses adipogenic differentiation in healthy cardiomyocytes, and ACM cardiomyocytes showed reduced DLK1 levels and thus promoted adipogenic differentiation [100].

Fibroblast–cardiomyocyte interactions occur via Cx43-dependent coupling and paracrine pro-fibrotic signaling [101]. Single-cell RNA sequencing has identified the TGF-β ligand–receptor interaction as a common cascade across cardiac cell types [102]. As described in Section 4.1, NFκB activation recruits CCR2^+^ macrophages that release pro-inflammatory cytokines, activating cardiac fibroblasts and exacerbating myocardial injury [12,103].

Epicardial cells additionally contribute by transdifferentiating into fibro-adipogenic progenitors [104,105] via pathways described in Section 4.2 and Section 4.4. These intercellular networks create self-perpetuating cycles of structural and electrical remodeling underlying progressive arrhythmic substrates in ACM.

Beyond traditional immune cell populations, disease-associated remodeling extends to extra-cardiac and neuronal cells. Bone marrow-derived mesenchymal stromal cells (BM-MSCs) exhibited enhanced migration to the hearts of *Dsg2*^mut/mut^ mice, where they heavily promoted alterations in cytoskeletal organization [61]. Additionally, as stated above, cardiac sympathetic neurons expressing desmosomal gene variants exhibited decreased axonal sprouting alongside abnormal varicosity patterning in ACM subjects. This neuronal remodeling provides an independent, early-stage substrate for stress-induced arrhythmias [62] and serves as an additional reminder of the importance of germline mutations in disease modeling as opposed to cardiomyocyte-specific KO models.

Lastly, topological singularities, which are specific points of continuity interruption, have been proposed as a mechanism of myocardial injury and could merit investigation in ACM [106].

## 5. Immunomodulatory Therapies in ACM

**Figure 4:**
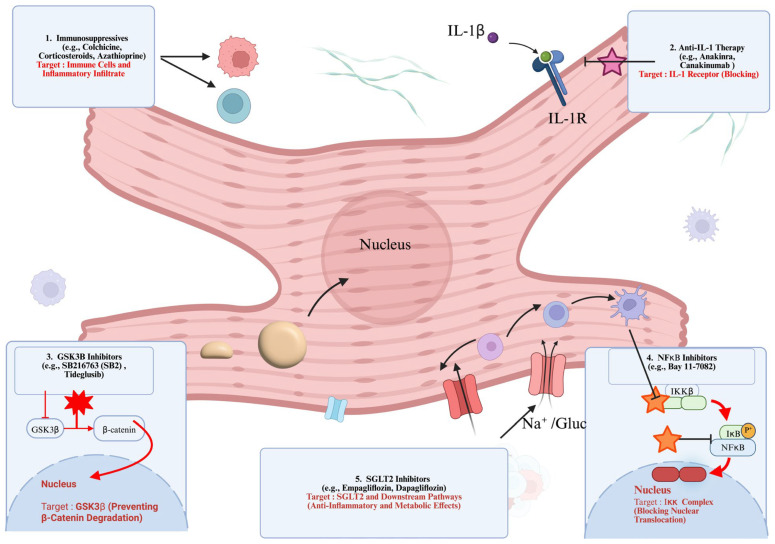
Therapeutic targets in ACM-associated inflammation. Overview of emerging pharmacological strategies targeting inflammatory and molecular pathways in ACM. (1) Immunosuppressives (e.g., colchicine, corticosteroids, azathioprine) reduce immune cell infiltration. (2) Anti-IL-1 therapies (e.g., anakinra, canakinumab) block IL-1 receptor signaling. (3) GSK3β inhibitors (e.g., SB216763, Tideglusib) prevent β-catenin degradation, preserving Wnt signaling. (4) NFκB inhibitors (e.g., Bay 11-7082) block Iκκ complex activation and prevent nuclear translocation of NFκB. (5) SGLT2 inhibitors (e.g., empagliflozin, dapagliflozin) exert anti-inflammatory and metabolic effects via SGLT2 and downstream pathway modulation.

Since 1982, numerous advancements in our understanding of ACM pathogenesis have shifted therapeutic considerations away from an exclusive focus on arrhythmia suppression and heart failure management toward strategies that target the inflammatory substrate itself. Immunomodulatory interventions in ACM are therefore increasingly viewed as potential disease-modifying therapies, with the objective of limiting immune-mediated myocardial damage (i.e., pro-inflammatory), facilitating tissue repair (i.e., pro-resolving), and delaying fibrotic replacement and ventricular dysfunction (Figure 4) [40,45,57].

At the same time, advances in genotype-informed therapies aim to correct or mitigate the effects of DGVs that underlie ACM. Given the interplay between genetic susceptibility and inflammatory activation, future treatment paradigms may involve the integration of molecularly targeted and immune-directed approaches to more effectively alter disease trajectory.

Myocardial inflammation in ACM may also have important implications for arrhythmia management. Inflammatory remodeling is associated with altered ion channel expression, disruption of cell–cell coupling, and increased electrical instability, all of which can dynamically influence arrhythmic risk. As observed in inflammatory cardiomyopathies, VAs, occurring in the setting of active myocardial inflammation, may demonstrate reduced responsiveness to standard rhythm control strategies until inflammatory activity is suppressed [107,108]. Consequently, controlling inflammation may represent an adjunctive strategy in ACM-related arrhythmia management, enhancing responsiveness to pharmacological therapy and improving outcomes following catheter ablation but not currently replacing the need for an ICD in patients at high risk [57,91,107,108].

### 5.1. Immunosuppressive Drugs

The rationale for broad-acting immunosuppressive strategies mainly relies on the evidence of inflammatory lymphomonocytic infiltrates in ACM patients. Additionally, the presence of circulating autoantibodies represents a therapeutic target in ACM, and, as in myocarditis, anti-heart and anti-intercalated disc antibody positivity may help to predict the response to immunosuppressive therapy [109,110]. Regimens based on corticosteroids in combination with azathioprine or mycophenolate mofetil have long been used in the treatment of autoimmune myocarditis and inflammatory dilated cardiomyopathy by improving left ventricular systolic function and reducing VA burden [107,111]. Similar benefits have been reported in selected patients with ACM. Case series describe favorable responses to immunosuppression in patients with biopsy-proven myocarditis carrying DGVs, particularly during *DSP*-related “hot phases”, where treatment may reduce chest pain and VAs, likely through attenuation of myocardial inflammation and edema [45,57,112].

Conventional immunosuppression in ACM populations appears to be safe, provided the exclusion of clinical contraindications, including active myocardial viral infection [45,57].

### 5.2. Pro-Inflammatory Cytokines and Directed-Signaling Pathway Drugs

Targeting pro-inflammatory cytokines aims to interrupt upstream specific signaling cascades implicated in ACM. Among these, inhibition of IL-1 has been the most extensively explored. Anakinra has demonstrated improvements in symptoms and systolic function in myocarditis and inflammatory cardiomyopathy [113,114,115]. In the setting of ACM, IL-1 inhibition has been employed on an individualized basis, particularly in patients with *DSP*-related ACM with recurrent inflammatory flares, with promising effects [45,57]. The role of inhibition of other pro-inflammatory cytokines, such as IL-6 and TNFα, remains unexplored.

The NFκB signaling axis represents a central regulator of inflammatory responses implicated in ACM, and its inhibition by Bay11-7082 attenuated ACM phenotypes in preclinical models [11,12]. Direct pharmacologic inhibition of NFκB has not reached clinical application because of unacceptable off-target effects; furthermore, colchicine, an indirect modulator of NFκB signaling, was not effective in ACM [57].

Dysregulation of GSK3β, involved in the suppression of the Wnt/β-catenin pathway, has also been implicated in ACM pathogenesis, particularly in the promotion of fibrosis and inflammation, and is effectively inhibited by SB216763 in preclinical ACM models [60,74,116,117]. Tideglusib, a repurposed GSK3β inhibitor, has shown promise in experimental settings and is currently being tested in a Phase 2 trial (NCT06174220) [76].

The inhibition of CD14, a TLR4 co-receptor, in preclinical ACM models switched from a pro-inflammatory and pro-fibrotic phenotype to a pro-reparative one. Atibuclimab, an anti-CD14 monoclonal antibody, is currently being evaluated for ACM in a phase 1b trial (NCT06275893). Additionally, targeting inflammatory cell recruitment is another potential therapeutic avenue. Inhibition of CCR2^+^ monocyte trafficking may limit macrophage-driven myocardial injury in ACM [12].

### 5.3. Additional Therapeutics

Sodium-glucose cotransporter-2 inhibitors (SGLT2i) are a cornerstone in heart failure treatment and displayed anti-inflammatory and anti-fibrotic properties in an ACM mouse model via normalization of HIF-2α signaling [118], but specific evidence is lacking in patients with ACM. High-dose statins may also modulate oxidation, inflammation, and autonomic activation, altering disease progression in ACM patients (NCT06922994) [119].

### 5.4. Gene Therapy

In parallel with immune-targeted strategies, gene therapy approaches aimed at correcting the underlying molecular defects in ACM are advancing. Early human studies targeting *PKP2* are ongoing (NCT06228924, NCT06109181, and NCT05885412), with encouraging preliminary findings [120]. Experimental approaches to enhance gap junction integrity, such as delivery of the truncated Cx43 isoform, GJA1-20k, and other strategies, have improved electrical coupling and reduced arrhythmias in ACM models [96,121]. Delivery of fibroblast growth factor 21 (FGF21) mitigated the phenotype in *PKP2* ACM models [122]. While these strategies do not directly suppress inflammation, they address downstream consequences of inflammatory signaling and desmosomal dysfunction and may prove synergistic when combined with anti-inflammatory therapies. Challenges remain, including vector specificity, immune responses to adeno-associated viruses, and rare reports of gene therapy-associated myocarditis [123,124].

## 6. Conclusions

ACM is increasingly understood as a disorder in which inflammatory signaling plays a central and active role alongside inherited defects in desmosomal and non-desmosomal genes. Inflammation influences not only clinical presentation but also arrhythmic risk, structural progression, and long-term outcomes. Genetic predisposition and immune activation are closely intertwined in ACM, with cardiomyocyte-intrinsic abnormalities creating a permissive environment for inflammatory injury that, in turn, accelerates myocardial damage and electrical instability.

Insights from immunogenetic research have clarified how pathogenic variants associated with ACM can engage inflammatory pathways, including maladaptive NFκB and GSK3β signaling and T cell responses, directly within the myocardium. These discoveries expand the traditional concept of a final common pathway in ACM, positioning immune dysregulation as a convergent mechanism that interacts with mechanical failure and arrhythmogenic remodeling. Clinically, this evolving framework supports a broader therapeutic approach in ACM, in which antiarrhythmic and heart failure therapies may be complemented by immunomodulatory strategies in carefully selected patients with evidence of active inflammation.

The paradigm of ACM as an inflammatory disease has some limitations: (i) myocardial inflammation is not homogeneously detected in all preclinical models and patients with ACM; (ii) several other molecular mechanisms contribute to ACM onset and progression; and (iii) prospective trials are needed to formally assess the safety and efficacy of immunosuppressive and molecular targeted therapies in inflammatory ACM, which cannot currently replace antiarrhythmic drugs and ICD.

Looking forward, comprehensive evaluation of ACM is likely to integrate genetic testing with immunologic profiling, including circulating cytokines, chemokines, autoantibodies, and inflammatory immune cells, to improve phenotypic classification and risk stratification. Such an approach could help identify patients with transient inflammatory phenotypes, those who may benefit from targeted immunosuppression, and those requiring early referral for advanced interventions.

## Figures and Tables

**Table 1:** Genes associated with ACM utilizing ClinGen-curated and guideline-reported evidence.

Section 1 and Section 2: ClinGen ARVC Gene Curation Expert Panel ^2^						
Gene	Gene Name	Protein Class	Inheritance	Predominant Ventricular Phenotype	Frequency ^1^	Classification
DEFINITIVE (ClinGen Classification) ^2^						
*PKP2*	Plakophilin-2	Desmosomal	AD	Right-dominant (ARVC)	20–46% ^1^	Definitive
*DSP*	Desmoplakin	Desmosomal	AD (rarely AR)	Left-dominant (ALVC)/biventricular	10–15% ^1^	Definitive
*DSG2*	Desmoglein-2	Desmosomal	AD	Biventricular (frequent LV involvement)	5–10% ^1^	Definitive
*DSC2*	Desmocollin-2	Desmosomal	AD (rarely AR)	Right-dominant (ARVC)	2–5% ^1^	Definitive
*JUP*	Junction Plakoglobin	Desmosomal	AD/AR	Biventricular (Naxos disease if AR)	<2% ^1^	Definitive
*TMEM43*	Transmembrane Protein 43	Nuclear Envelope	AD	Right-dominant/biventricular	1–2% ^1^	Definitive
MODERATE (ClinGen Classification) ^2^						
*DES*	Desmin	Intermediate Filament	AD/AR	Biventricular (conduction disease common)	<1% ^1^	Moderate
*PLN*	Phospholamban	Sarcoplasmic Reticulum	AD	Left-dominant/biventricular (DCM overlap)	<1% ^1^	Moderate
Section 3: HRS 2019 Expert Consensus & 2023 ESC Cardiomyopathy Guidelines ^3,4^						
Gene ^†^	Gene Name	Protein Class	Inheritance	Predominant Ventricular Phenotype	Frequency ^1^	Classification
LIMITED: HRS 2019 & ESC 2023; not ClinGen-classified ^†^						
*FLNC*	Filamin C	Cytoskeletal/Sarcomere	AD	Left-dominant (NDLVC/ALVC); ESC 2023: classified under NDLVC	<1% ^1^	Limited
*RBM20*	RNA-Binding Motif Protein 20	RNA Splicing Factor	AD	Biventricular/left-dominant (DCM/NDLVC); ESC 2023: classified under DCM + NDLVC	<1% ^1^	Limited
*LMNA*	Lamin A/C	Nuclear Lamina	AD	Biventricular (DCM overlap); ESC 2023: classified under DCM + NDLVC	<1% ^1^	Limited
*SCN5A*	Nav1.5 (Voltage-Gated Na^+^ Channel)	Ion Channel	AD	Left-dominant; James et al. 2021 [19] not in ARVC column of ESC 2023	<1% ^1^	Limited
*CDH2*	N-Cadherin (Cadherin-2)	Adhesion	AD	Right-dominant (ARVC)	<1% ^1^	Limited
*CTNNA3*	αT-Catenin	Adhesion	AD	Right-dominant (ARVC)	<1% ^1^	Limited
*TJP1*	Tight Junction Protein 1 (ZO-1)	Tight Junction	AD	Right-dominant (ARVC)	<1% ^1^	Limited

^1^ Frequency reflects proportion among desmosomal gene variant ACM cases only. Estimates derived from Hall CL et al. *Eur J Hum Genet.* 2018; 26: 1312–1318 [10]. ^2^ Evidence strength (definitive/moderate) per ClinGen ARVC Gene Curation Expert Panel: James CA et al. *Circ Genom Precis Med.* 2021; 14: e003273. PMID: 33831308 [19]. ^3^ Towbin JA et al. 2019 HRS Expert Consensus Statement on Evaluation, Risk Stratification, and Management of Arrhythmogenic Cardiomyopathy. *Heart Rhythm.* 2019; 16: e301–e372 [20]. ^4^ Arbelo E et al. 2023 ESC Guidelines for the Management of Cardiomyopathies. *Eur Heart J.* 2023; 44: 3503–3626 [21]. ^†^ Section 3 genes are NOT classified by ClinGen for ARVC. They are reported as candidate or associated genes in the HRS 2019 and/or ESC 2023 guideline documents. FLNC, RBM20, and LMNA are classified under NDLVC or DCM phenotype categories in ESC 2023, not under ARVC. SCN5A is referenced in Towbin 2019 [20] as an ACM-associated gene but does not appear in the ARVC column of ESC 2023. ACM, arrhythmogenic cardiomyopathy; AD, autosomal dominant; AR, autosomal recessive; ALVC, arrhythmogenic left ventricular cardiomyopathy; ARVC, arrhythmogenic right ventricular cardiomyopathy; ClinGen, Clinical Genome Resource; DCM, dilated cardiomyopathy; ESC, European Society of Cardiology; HRS, Heart Rhythm Society; NDLVC, non-dilated left ventricular cardiomyopathy.

## Data Availability

No new data were created or analyzed in this study.
